# POLICIES FOR PRESERVING THE WORK ACTIVITY OF PEOPLE IN RETIREMENT AGE IN HIGHER EDUCATION INSTITUTIONS IN BULGARIA

**DOI:** 10.1093/geroni/igz038.2402

**Published:** 2019-11-08

**Authors:** Zaharina A Savova

**Affiliations:** Medical College Yordanka Filaretova at Medical University - Sofia, Sofia, Bulgaria

## Abstract

Demographically, aging of the world’s population is a fact that requires the implementation and development of integrated, active policies for the inclusion of older people in economic and social life. In response to this challenge, an international UN plan for active aging was adopted in Madrid in 2002. By adopting this plan, all EU member states are committed to contribute to active aging by integrating the rights and needs of older people into national economic and social policies. The European Commission formulates the concept of active aging as adopting a healthy lifestyle, longer labor market participation, later retirement, and retention after retirement. In recent years managers of higher education institutions in Bulgaria have been pursuing a policy of preserving the labor and intellectual potential of retired research and teaching staff. There are different practices and activities aimed at preserving this valuable resource built for decades. This paper outlines the main activities and evaluation of the effectiveness of the Mentoring Program for Shared Experience and Knowledge. This pilot program was implemented over a five-year period from 2012 to 2017. It involved teachers in pre-retirement and retirement age working in higher education institutions. Monitoring and evaluation of the different forms of activities included in the program were made. The benefits for all participants have been derived. The results and the analysis made support the initial suggestion that the program is an opportunity for gradual withdrawal from work activity, preserving social well-being and successful adaptation to retirement from active labor activity.

